# What Cure Models Can Teach us About Genome-Wide Survival Analysis

**DOI:** 10.1007/s10519-015-9764-0

**Published:** 2015-11-09

**Authors:** Sven Stringer, Damiaan Denys, René S. Kahn, Eske M. Derks

**Affiliations:** Department of Complex Trait Genetics, Center for Neurogenomics and Cognitive Research (CNCR), Neuroscience Campus Amsterdam (NCA), VU Amsterdam, Amsterdam, The Netherlands; Department of Psychiatry, Academic Medical Center, Amsterdam, The Netherlands; Department of Psychiatry, Rudolf Magnus Institute of Neuroscience, University Medical Center, Utrecht, The Netherlands

**Keywords:** Proportional hazards model, Logistic regression, Cox regression, Accelerated failure time model, Simulation study

## Abstract

The aim of logistic regression is to estimate genetic effects on disease risk, while survival analysis aims to determine effects on age of onset. In practice, genetic variants may affect both types of outcomes. A cure survival model analyzes logistic and survival effects simultaneously. The aim of this simulation study is to assess the performance of logistic regression and traditional survival analysis under a cure model and to investigate the benefits of cure survival analysis. We simulated data under a cure model and varied the percentage of subjects at risk for disease (cure fraction), the logistic and survival effect sizes, and the contribution of genetic background risk factors. We then computed the error rates and estimation bias of logistic, Cox proportional hazards (PH), and cure PH analysis, respectively. The power of logistic and Cox PH analysis is sensitive to the cure fraction and background heritability. Our results show that traditional Cox PH analysis may erroneously detect age of onset effects if no such effects are present in the data. In the presence of genetic background risk even the cure model results in biased estimates of both the odds ratio and the hazard ratio. Cure survival analysis takes cure fractions into account and can be used to simultaneously estimate the effect of genetic variants on disease risk and age of onset. Since genome-wide cure survival analysis is not computationally feasible, we recommend this analysis for genetic variants that are significant in a traditional survival analysis.

## Introduction

In the last decade many genome-wide association (GWA) studies have been published. The GWAS catalog currently contains 1924 GWA studies (www.genome.gov/gwastudies, accessed June 27, 2014) (Welter et al. 2014). For example, in the field of psychiatry, the Psychiatric Genomics Consortium (PGC) has reported GWA analyses on diseases such as ADHD (Neale et al. 2010), bipolar disorder (Sklar et al. 2011), major depressive disorder (Ripke et al. 2012), and schizophrenia (Schizophrenia Working Group of the Psychiatric Genomics Consortium 2014). These studies typically include thousands or tens of thousands of subjects with the aim of identifying genetic variants that affect the risk of developing a disorder. Alternatively, researchers have aimed to identify genetic variants that affect age of onset of a disease. For example, Bergen et al. (2014) investigated which genetic variants affect age of onset in 2762 schizophrenia patients but did not find genome-wide significant SNPs, possibly due to lack of power. Identifying genetic variants which contribute to disease risk and age of onset are both legitimate research goals for which different analysis methods are typically applied. Risk of disorder is typically analyzed using logistic regression, while time to onset is often analyzed using survival analysis. In previous studies, the focus has been on the analysis of either disease risk or age of onset as separate outcome measures, while in reality a genetic variant may influence both types of outcome. In this study, we will evaluate the possibility of incorporating genetic effects on disease risk and age of onset within a single analysis using data simulation. We aim to investigate the implications of performing a traditional analysis in which genetic variants are assumed to show only a single type of genetic effect, while both types of effects are in fact present. Results will be compared with those of a more complex type of survival analysis, allowing for simultaneous estimation of disease risk and age of onset effects.

As the name implies, survival analysis can be applied to analyze survival times or, equivalently, age of death. However, the event of interest need not be death, but can be of any type, including timing of disease onset and disease relapse. In genetic survival analysis we may be interested in estimating genetic effects on time of disease onset. This type of analysis addresses the question whether risk allele carriers develop a disease at an earlier age than non-carriers.

For many diseases, the majority of subjects in a sample will not develop the disease during their lifetime. The fact that some subjects may never be affected by the disease of interest poses a problem for survival analysis. Traditional survival analysis treats all unaffected subjects similarly. However, there may be a qualitative difference between subjects that have not yet experienced the event and those that never will. Cure models can accommodate this by modelling disease risk (i.e., lifetime affected vs. lifetime unaffected) with a logistic model and only for lifetime affected cases the time to event is modeled with a survival model (Othus et al. 2012). The name ‘cure’ refers to the original development of the model in the context of long-term survival of cancer patients after treatment. In that context cure models explicitly model a subset of cured cancer patients, called the cure fraction, that die at ages more similar to healthy people compared to patients who are not cured (Prasad 2014). However, cure models can be applied to any context in which a subset of subjects is not affected by the event of interest. In this article, a cure model assumes populations are a mixture of subjects who will never develop the disorder of interest and those who will. In this case the proportion of subjects who will never develop the disorder would be the ‘cure’ fraction as for this group survival effects and time to disease onset are not applicable.

In this simulation study we apply the cure model to introduce both logistic and survival effects for a genetic variant. We investigate the bias in parameter estimation when performing a traditional survival analysis while including two types of genetic effects in the simulation model. Additionally, we investigate the implications of ignoring genetic background risk on the estimates. Heritable phenotypes often show a complex genetic architecture, meaning that many genetic variants contribute to the phenotype (Gratten et al. 2014). Since genetic variants are typically analyzed one at a time, the genetic background risk from other genetic variants is ignored. This can result in biased effect estimates (Gail et al. 1984; Stringer et al. 2011). Therefore we will simulate cure model data with and without the presence of genetic background risk.

In this study we aim to address the following two research questions. The first question regards the influence of a cure fraction on parameter estimation in traditional logistic analysis and survival analysis. The second question concerns the feasibility of analyzing data using the extended cure survival model. We will investigate whether the cure model offers advantages over traditional survival analysis. To answer these questions we look at several characteristics of the analysis models: test characteristics such as type-1 error rate and power, bias in the effect size estimates, sensitivity to ignored genetic background risk, and practical considerations such as running time of the fitting procedure.

## Methods

### Simulation model

Data were simulated according to a cure model with a logistic component and a survival component. The logistic component models the probability of being affected by disease. It divides the population in two distinct subpopulations: subjects at risk and subjects not at risk. The proportion of subjects not at risk is the cure fraction. Cure fractions range from 0 to 1, with 1 representing a situation in which no subject will be affected during their lifetime and 0 representing a situation in which all subjects will become affected eventually. While a cure fraction of 1 is not very useful to consider, because there would be no variation in the data, a cure fraction of 0 is effectively a traditional survival model, since all subjects will in principle develop the disease during their lifetime. In that case the logistic part of the model is not defined. The cure fraction is mostly determined by the intercept of the logistic model. An intercept of minus infinity (or simply an extremely large negative value) corresponds to a cure fraction of zero. In that case all subjects are at risk. The logistic effect parameter (i.e., the log(odds ratio)) models the contribution of a risk allele to disease risk.

On the other hand, the survival component of the cure model models the age of onset for subjects who will develop the disease. A popular survival model is the Cox proportional hazard (PH) model. The Cox PH model assumes that the hazard ratio is constant over time. The hazard is a measure of how likely it is that an event will happen at a particular time given that it has not yet happened. For example, if 0.9 % of people die exactly at age 60 and 90 % of people die at 60 or older, then the hazard of dying at age 60 is approximately 0.9 %/90 % = 1 %. Although the hazard of dying may change over time, the proportional hazards assumption implies that the ratio of hazards between risk allele carriers and non-carriers is constant over time. The Cox PH model is elegant in that it does not make any assumptions about the hazard function itself, but only about the proportion of two hazard functions.

Although the Cox PH model is useful for analyzing survival data, it cannot be used to simulate data as it is a semi-parametric model and does not specify the hazard function. Therefore we have chosen the parametric Weibull accelerated failure time (AFT) model to model age of onset in our cure model, since it meets the proportional hazard assumption of the Cox PH model (Kleinbaum and Klein 1996). Moreover, the AFT parameter of the Weibull AFT model and the hazard ratio of the Cox PH model provide different interpretations on the same survival effect, since both parameters can be converted into each other. Therefore we can choose a hazard ratio, convert it into an AFT parameter and use the Weibull AFT model to simulate age at event for subjects who are at risk. The Weibull AFT model has three parameters: the survival SNP effect size and a scale and rate parameter of the Weibull distribution. Depending on the interpretation, the survival SNP effect size models the contribution of a risk allele to the hazard [log(hazard ratio)] or the expected age of initiation [log(AFT parameter)]. The scale and rate parameter of the Weibull distribution determines the distribution of age of onset for subjects carrying no risk allele at that SNP. Since we will not study the effect of the scale and rate parameters, we arbitrarily fixed both parameters at twenty. This corresponds to a median age of onset of 19.6 years and a variance of 1.56 years.

For the SNP of interest (i.e., the SNP to be analyzed while ignoring all other background SNPs) we modeled two allelic effect size parameters: an odds ratio (OR) for the logistic allelic effect and a hazard ratio (HR) for the survival allelic effect. In addition to these SNP parameters, we also simulated a logistic and survival effect for genetic background risk. The genetic background risk is the cumulative genetic risk of all other SNPs that are not currently analyzed. Although we include the background risk as a covariate in our simulation model, we will omit the covariate in the analysis models to approximate a traditional GWA analysis, in which each SNP is analyzed independently, while ignoring other SNPs. In the simulation model, the genetic background risk is normally distributed with mean zero. In other words, an individual with a genetic background risk of zero represents an average amount of genetic background risk. In our simulations, we set the background heritability to 0 % (no background risk) or 50 % (background risk) respectively, for both logistic and survival effects. The background heritability is defined as:

$$h_{bg}^{2} = \frac{{\beta_{bg}^{2} Var\left( {X_{bg} } \right)}}{{\beta_{bg}^{2} Var\left( {X_{bg} } \right) + Var\left( \varepsilon \right)}}$$

where *Var* is variance, ε is the error defined on a linear scale (log(OR) or log(age of onset) respectively), β_bg_ is the background effect size, and X_bg_ is a random variable representing the genetic background risk.

To facilitate comparison of SNP effect sizes, we define the SNP heritability as:

$$h_{SNP}^{2} = \frac{{\beta_{SNP}^{2} Var\left( {X_{SNP} } \right)}}{{\beta_{SNP}^{2} Var\left( {X_{SNP} } \right) + \beta_{bg}^{2} Var\left( {X_{bg} } \right) + Var\left( \varepsilon \right)}}$$

where SNP is the SNP to be analyzed, β_SNP_ its effect size on a linear scale [log(OR) or log(age at event) respectively], and X_SNP_ a random variable representing the number of risk alleles present. In the model we set Var(X_bg_) = 37,500 and Var(X_SNP_) = 0.375, corresponding to allele frequencies of 0.25 and 100,000 background SNPs. Finally, we set Var(ε) to the standard variance of the error distribution corresponding to the type of effect (i.e., logistic or survival). Fixing these parameters for all simulation models results in a one to one correspondence between effect size (i.e., OR and HR) and heritability.

Survival data typically involves censoring: the unavailability of age of onset for some subjects. For example, subjects who have not been affected by disease at the time of their assessment have an unknown age of disease onset and are therefore referred to as censored. An important assumption in survival analysis is uninformed censoring. This means that whether or not a subject is censored is unrelated to age of onset. In other words, censored subjects should on average develop the disease at the same time as uncensored subjects. We assumed uninformed censoring in our simulation model. To simulate censoring we assumed uniform right-censoring between the ages of 15 and 25 years. This interval covers the range of plausible values for age of onset in our simulation model. In other words, each subject drops out of the study randomly at an age between 15 and 25 years old. If, by that time, the subject has not contracted the disease of interest, he or she is censored and age of onset is unknown.

The above model results in the simulation of essential characteristics of survival GWA data with logistic and/or survival SNP effects, at varying cure fractions, and at a particular genetic background risk.

### Analysis model comparison

To investigate the implications of analyzing data using traditional survival or logistic analysis, while the data is in fact generated according to a cure model, we have simulated 10,000 data sets of 500 subjects for 152 parameter combinations. We varied the following parameters: (1) logistic SNP heritability [0, 1 %], (2) survival effect SNP heritability [0, 1 %], (3) background heritability for logistic and survival effects [0, 50 %], and (4) cure fraction 0–90 % with steps of 5 %. For each simulated data set, we have estimated the odds ratio using a logistic regression model and the hazard ratio using a semi-parametric Cox PH model. We will report type-1 error rate, power, and bias of effect sizes.

To investigate the value of using a cure analysis model to estimate the logistic and survival effect simultaneously, we have also analyzed a subset of the simulated data with a cure Cox PH model instead of the traditional Cox PH model. In the cure analysis a semi-parametric PH mixture cure model was fitted using the expectation–maximization (EM) algorithm (Dempster et al. 1977). Since the lifetime disease status of currently healthy subjects is unknown, this status can be considered a latent variable. In the EM algorithm parameters are first initialized arbitrarily and then refined iteratively in a two-step approach. First the expected cure status (the latent variable) of all subjects is computed based on the current model parameter estimates (E-step). This expected cure status is subsequently used to readjust the cure model parameters by maximizing the (partial) log likelihood of the traditional Cox survival model (M-step). Although this procedure is guaranteed to converge to a local optimum, convergence of the EM algorithm in mixture models can be relatively slow. For further details about the estimation method used we refer to Cai et al. (2012).

Since fitting the cure Cox PH model was computationally demanding, we restricted the analysis to the following 20 parameter combinations: (1) logistic and survival SNP heritability both 0 % or 1 %, (2) background heritability 0 % or 50 %, and (3) cure fraction [10, 30, 50, 70, 90 %]. In addition to type-1 error, power, and bias, we will also report the running time for the cure PH analysis, since this type of analysis is expected to be computationally more demanding.

All results are reported as median values based on 10,000 simulations. We used a significance threshold of 0.1 instead of the traditional threshold of 0.05 to increase the reliability of the type-1 error rate estimates. This choice does not affect our qualitative conclusions. All statistical analyses were performed in R version 3.1.0 using the glm, survival, and smcure packages respectively (R Core Team 2012). Data simulation was performed in R as well.

## Results

### Traditional analysis of cure data

#### Type 1 error

**Fig. 1:**
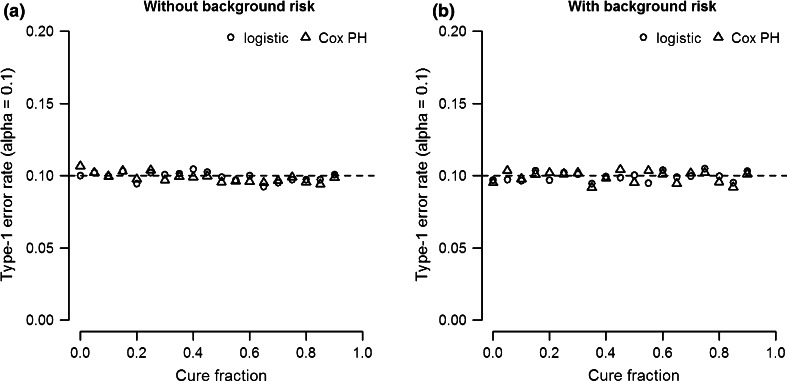
Type-1 error rate (alpha = 0.1) as function of cure fraction for logistic and Cox PH analysis coefficients. **a** Simulation data without background heritability. **b** Simulation data with 50 % background heritability for both logistic and survival effects. The SNP heritability of logistic and survival effect is 0 %

The type-1 error rate of both logistic regression and Cox PH analysis is controlled at the specified alpha level. Figure 1 shows the type-1 error rate (alpha = 0.1) as a function of cure fraction. Figure 1a shows results based on a simulation model without genetic background risk (0 %), while Fig. 1b is based on a model with 50 % background heritability for both the logistic and survival effect. The results indicate that neither the introduction of cure fractions nor genetic background risk inflate the type-1 error rate of logistic and Cox PH analysis.

#### Power

**Fig. 2:**
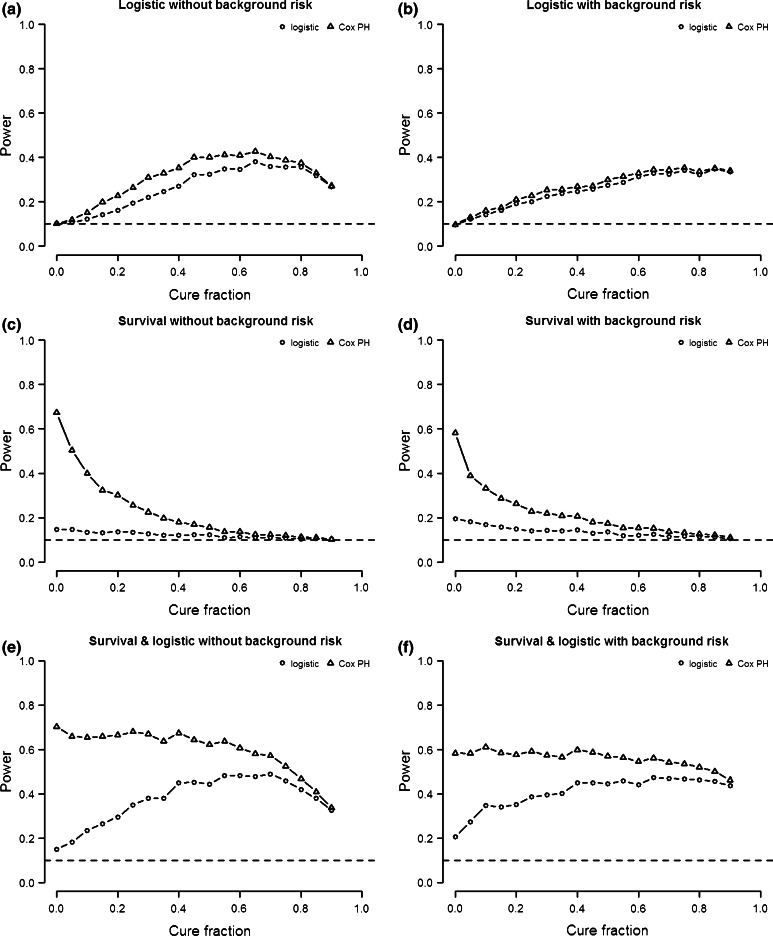
Power as function of cure fraction for logistic and Cox PH analysis. **a**, **b** Logistic SNP heritability 1 %. **c**, **d** Survival SNP heritability 1 %. **e**, **f** Logistic and survival SNP heritability 1 %. *Left* simulation data without background heritability. *Right* simulation data with 50 % background heritability for both logistic and survival effect. *Horizontal line* represents type-1 error rate (0.1)

The power to detect either type of genetic effect with traditional logistic and survival analysis depends on the types of genetic effect that are present in the simulated cure data. We distinguish three types of genetic variants: (1) variants with only a logistic effect, (2) variants with only a survival effect, and (3) variants with both a logistic and a survival effect. Figure 2 shows the power of logistic regression and Cox PH analysis as a function of cure fraction for each of these three combinations of genetic effects. As mentioned before, a cure fraction of 0 corresponds to a traditional survival model where no logistic effect is defined, while a cure fraction of 1 corresponds to a situation in which none of the subjects will ever contract the disease and neither a logistic effect nor a survival effect can be defined. We therefore focus mostly on cure fractions between 0 and 1.

The first type of variant only exhibits a logistic effect (Fig. 2a, b). If no genetic background risk is present, power initially increases with cure fraction, but decreases again as the cure fraction approaches 1 (Fig. 2a). This is true for both the logistic and the Cox PH analysis. Because the logistic effect is not defined at a cure fraction of 0, the power to detect a logistic effect starts at type-1 error rate. Note that the Cox PH analysis is sensitive to logistic effects, since no survival effect is actually present. In fact, the power of Cox PH analysis to detect logistic effects is higher than that of the logistic analysis, which can be explained by the fact that the latter does not account for uninformed censoring.

The results in the previous paragraph are based on a simulation model without genetic background risk. When we introduce genetic background heritability (50 %) in the model, the power profile changes in a similar way for the Cox PH and logistic analysis (Fig. 2b). First of all, the difference in power between the two analyses decreases. Furthermore, the introduction of genetic background risk decreases the power for both analyses for most cure fractions (<0.8).

The second model only exhibits a survival effect (Fig. 2c, d). If no genetic background risk is present (Fig. 2a), the power of the Cox PH analysis is optimal if the cure fraction is zero. Its power decreases and approaches the type-1 error rate as the cure fraction approaches one, because less and less subjects will ever experience the event. This is why data with a cure fraction close to one (i.e., very few subject ever experience the event) cannot be analyzed. Compared to Cox PH analysis, logistic analysis has much lower power, because no logistic effect is present. However, logistic analysis is somewhat sensitive to the survival effect, since the power is larger than the type-1 error rate at low cure fractions (<0.5).

The power curves of logistic and survival analysis are qualitatively similar for models with background heritability (50 %) (Fig. 2d). However, compared to models without genetic background risk, the power of survival analysis is lower, while that of logistic regression is higher.

The third type of model exhibits both a survival effect and a logistic effect of equal size (h^2^ = 1 %) (Fig. 2e, f). The power to detect either type of genetic effect is therefore a function of the power to detect logistic only effects and survival only effects The results indicate that in a model with no genetic background risk the power of Cox PH analysis remains high for cure fractions <0.5 (Fig. 2e). This can be explained by the fact that at low cure fractions (<0.25) the analysis is sensitive to logistic effects, while at higher (>0.25) cure fractions Cox PH analysis is sensitive to survival effects. On the other hand, the power of logistic analysis to detect either type of genetic effect is very similar to the power to detect a logistic only effect, since logistic analysis has low power to detect survival effect.

Again, the power curves for both types of analysis are qualitatively similar if we introduce background heritability (50 %) (Fig. 2d). Compared to models without genetic background risk, the power of survival analysis is lower in models with genetic heritability, while the power of logistic regression is higher.

#### Bias

**Fig. 3:**
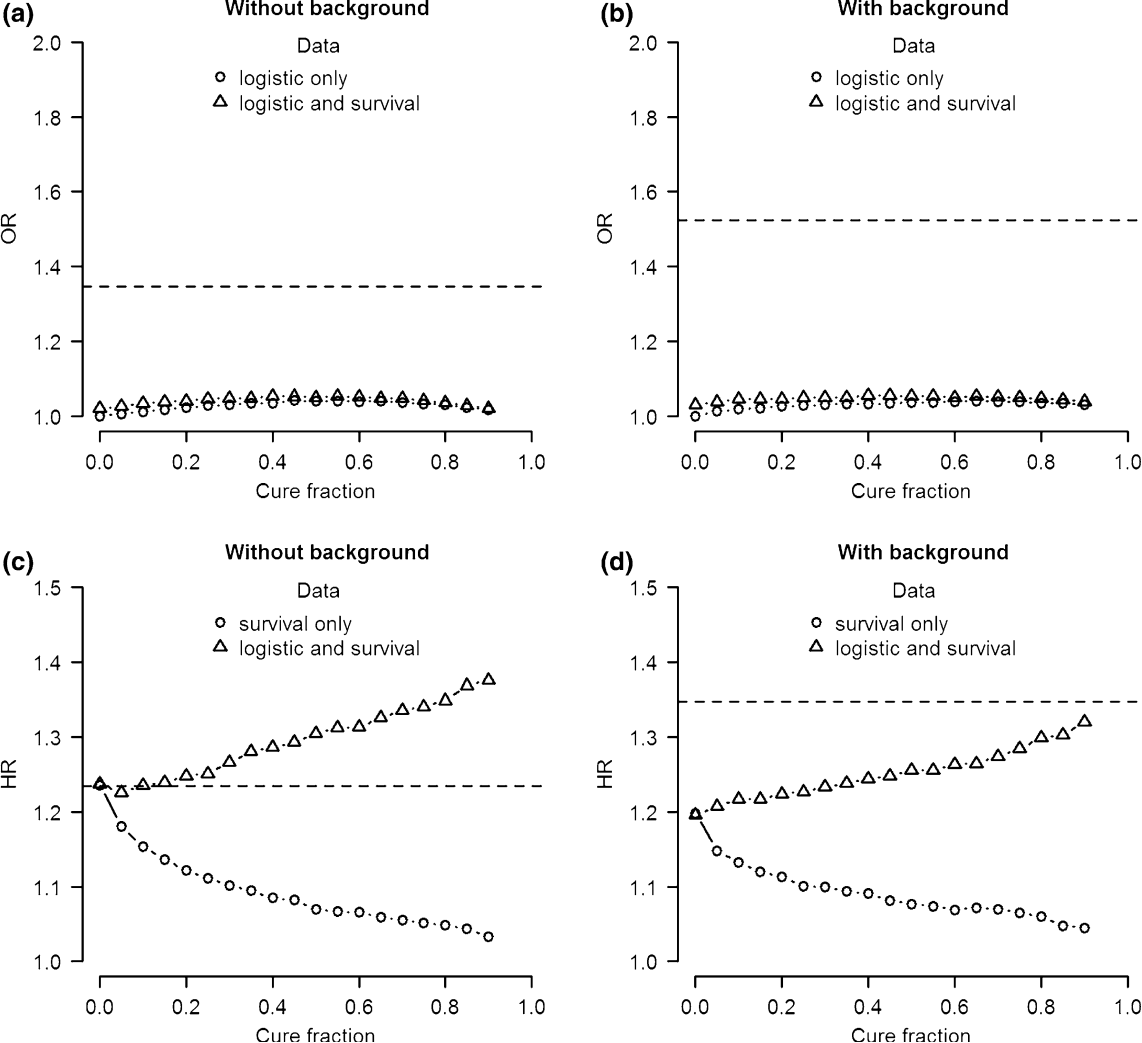
Median effect size estimate of 10,000 simulations as a function of cure fraction for logistic (**a**, **b**) and Cox PH analysis (**a**, **b**). *Left plots* simulation data without background heritability. *Right plots* simulation data with 50 % background heritability for both logistic and survival effect. The *horizontal line* represents the simulated effect size

We also investigated the bias in parameter estimates when performing logistic regression and Cox PH analysis with cure model data. First we compare the odds ratio estimate of the logistic analysis for data including only a logistic effect with the estimate for data with both a logistic and a survival effect. Figure 3a shows the median estimated odds ratio of 10,000 simulations as a function of cure fraction if no genetic background risk is present. We consider two situations: analyzing simulated data including only a logistic effect and analyzing simulated data including both a logistic and a survival effect. The estimated odds ratio in both situations slightly increases with cure fraction at first (cure fractions <0.5), before it decreases again as the cure fraction approaches 1 (cure fractions >0.5). However, in both situations the odds ratio is greatly underestimated. Again this is due to the fact that a logistic analysis does not account for uninformed censoring. Since all censored cases are considered controls in the logistic analysis, the logistic effect is underestimated. This underestimation is somewhat less if the analyzed genetic variant exhibits both a logistic and a survival effect.

A similar picture emerges if we introduce background heritability (50 %) into the model (Fig. 3b). The main difference is that the odds ratio used to simulate the data is larger. This is because in both models the explained variance of the logistic effect is set to 1 %. Therefore, increasing the background heritability results in an increase of the simulated odds ratio.

Next we compare the median hazard ratio estimates of the Cox PH analysis for simulated data including only a survival effect with estimates for simulated data including a survival effect and a logistic effect. The results in the absence of background heritability are shown in Fig. 3c. If only survival effects are present, the median estimated hazard ratio decreases with cure fraction. If the cure fraction is 0, the assumptions of the Cox PH analysis are met and the estimate of the hazard ratio is unbiased. However, as the cure fraction increases the assumption of uninformed censoring no longer holds, since clearly unaffected subjects will be more likely to be never affected by disease than affected subjects, resulting in increased bias. On the other hand, if both logistic and survival effects are present, the estimated hazard ratio increases with cure fraction. The sensitivity to detect the logistic effect not only offsets the underestimation, but even results in overestimation if the cure fraction is larger than 0.

The same qualitative pattern is observed for data with genetic background risk (Fig. 3d). However, due to an overall decrease of the estimated hazard ratio and an increase of the simulated hazard ratio, all analyses result in underestimation.

### Cure analysis of cure data

The bias in traditional analyses of cure survival data is caused by model misspecification. Alternatively, we could apply a cure survival analysis. Contrary to the traditional logistic and Cox PH analysis, the cure PH analysis simultaneously estimates both the odds ratio and the hazard ratio. In the following section we report the type-1 error, power, and bias in parameter estimation of a cure survival analysis compared to the traditional analyses.

#### Type 1 error

**Fig. 4:**
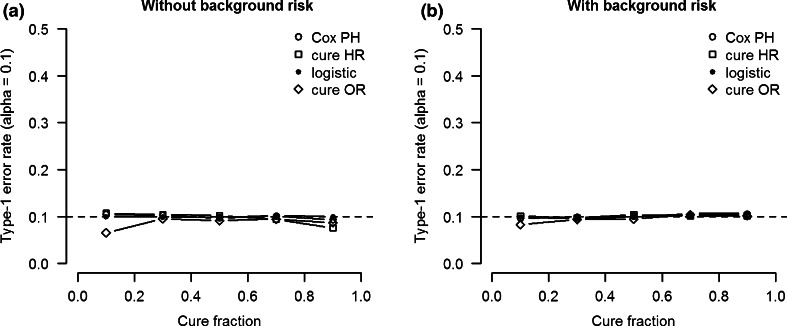
Type-1 error rate (alpha = 0.1) as a function of cure fraction for the cure model OR and HR coefficients compared to traditional logistic and Cox PH analysis coefficients. The SNP heritability of logistic and survival effect is 0 %. **a** Simulation data without background heritability. **b** Simulation data with 50 % background heritability for both logistic and survival effects

As in the traditional analyses, the type-1 error rate for the estimated odds ratio and hazard ratio of the cure PH model is controlled at the specified significance threshold (alpha = 0.1) for all cure fraction in models without background heritability (Fig. 4a). In fact the type-1 error rate for the estimated cure HR seems somewhat lower than that of the traditional estimates at cure fractions near 0 and 1. The type-1 error rate is closer to alpha for models in which background heritability is present for both the logistic and the survival effect (h^2^ = 50 %) (Fig. 4b).

#### Power

**Fig. 5:**
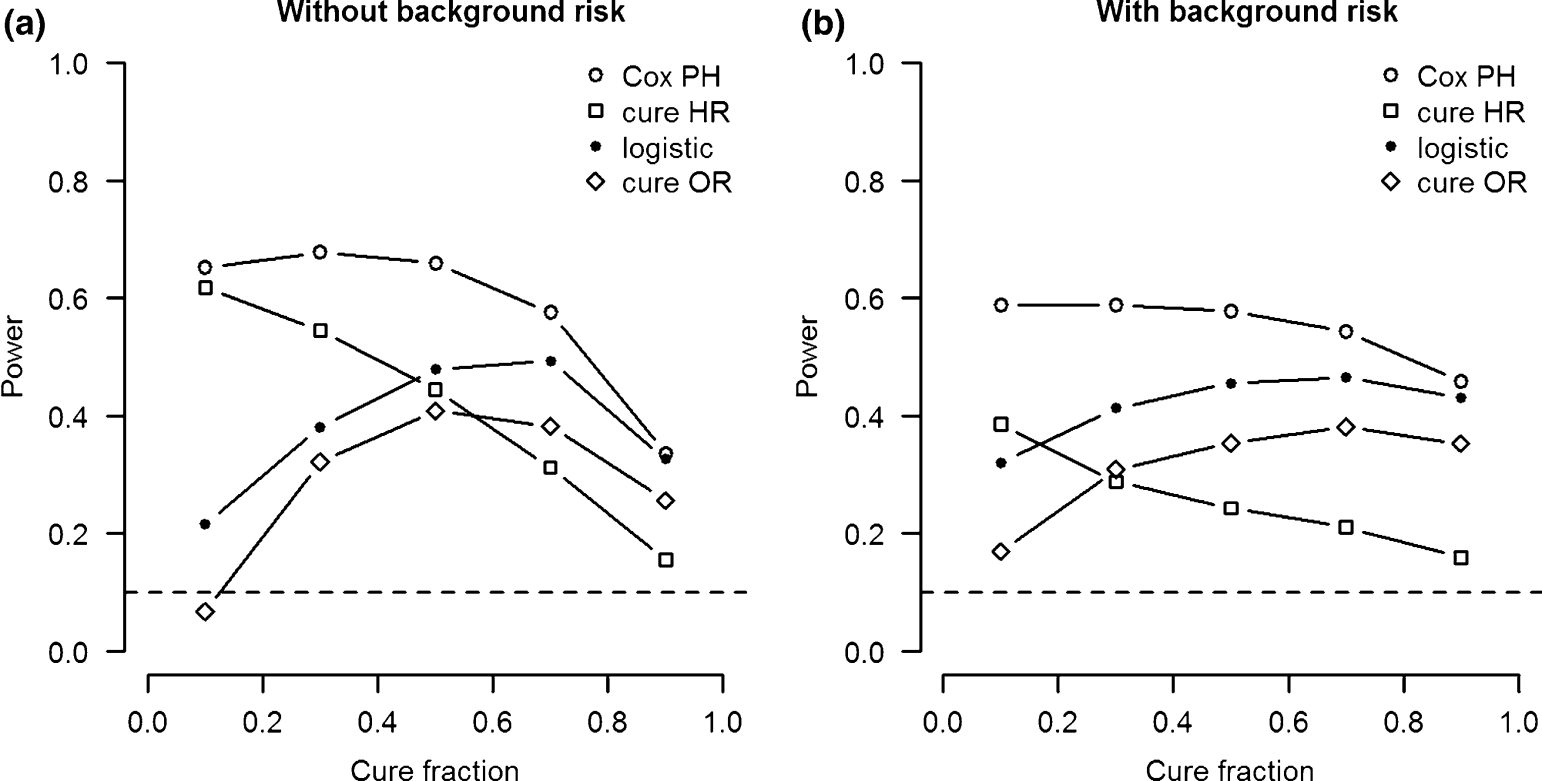
Power as function of cure fraction for cure model OR and HR coefficients compared to traditional logistic and Cox PH analysis. Logistic and survival SNP heritability 1 %. **a** Simulation data without background heritability. **b** Simulation data with 50 % background heritability for both logistic and survival effect. *Horizontal line* represents alpha level (0.1)

Figure 5a shows the power for all four estimates (i.e., Cox PH hazard ratio, cure hazard ratio, logistic odds ratio, and cure odds ratio) as a function of cure fraction in a model with both logistic and survival effects. The power curves for the cure model have a shape similar to the power curves of their respective traditional analyses. However, the power of the cure analysis is lower. The traditional analyses are sensitive to both types of effects, which results in increased power to detect either type of effect. In contrast, the two estimates of the cure PH model are only sensitive to the odds ratio or hazard ratio respectively. In other words, the power curves of the cure PH analysis provide a more accurate picture of the power to detect the type of effect corresponding to the type of effect size (i.e., OR or HR).

While there is no model misspecification for models with no background heritability, misspecification is a problem for models with genetic background heritability. Figure 5b shows that the power to detect a survival effect decreases considerably in models with 50 % background heritability compared to models without genetic background risk. This drop in power is larger for the cure PH analysis than for the traditional Cox PH analysis. On the other hand, the power to detect a logistic effect with a cure PH analysis increases if genetic heritability is introduced compared to a model without genetic background risk.

#### Bias

**Fig. 6:**
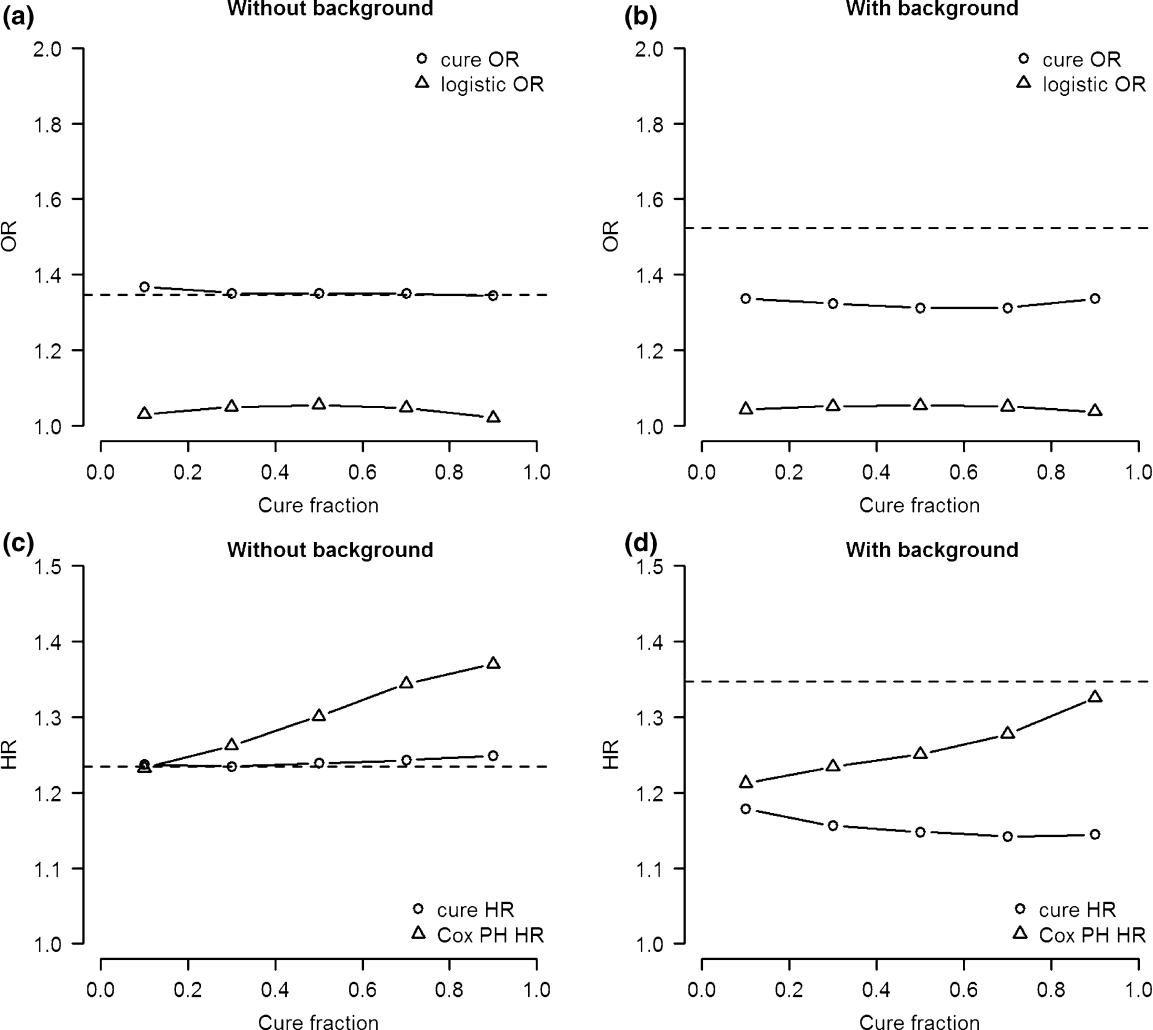
Median effect size estimate of 10,000 simulation of cure survival model and traditional model as a function of cure fraction for odds ratio (OR) (**a**, **b**) and hazard ratio (HR) (**a**, **b**). *Left panels* simulation data without background heritability. *Right panels* simulation data with 50 % background heritability for both logistic and survival effect. *Horizontal line* represents the simulated effect size

Finally, we compare the bias of traditional estimates and the cure PH estimates of the odds ratio and hazard ratio respectively. Figure 6a shows that the estimated odds ratio of the cure PH analysis is unbiased for a large range of cure fractions, while the estimate of the logistic analysis is severely underestimated as we have seen before. However, when we introduce genetic background risk the odds ratio of the cure PH analysis is underestimated as well, although not as much as the estimate of the logistic regression (Fig. 6b).

The estimate of the hazard ratio of the cure PH analysis is also relatively unbiased across cure fractions compared to the estimate of the Cox PH analysis if no genetic background risk is assumed. (Figure 6c). However, in the model with 50 % background heritability, both Cox PH analysis and cure PH analysis results in underestimated hazard ratio for varying cure fractions.

#### Analysis runtime

The fitting procedure for the cure PH model is computationally intensive. The median time over 10,000 simulations for fitting a cure PH model increased from 26 to 40 s as the cure fraction varied from 0.9 to 0.1 (data not shown). As the cure fraction approaches 0, convergence of the fitting procedure gets more difficult, since the odds ratio is not identified if the cure fraction is zero.

## Discussion

In this simulation study, we have compared different analysis methods to identify genetic effects in genome-wide survival data. Our first aim was to investigate the influence of a cure fraction on parameter estimation in traditional logistic analysis and survival analysis. Although logistic regression is typically used to detect genetic variants that affect disease risk, and Cox PH regression is used to detect variants that affect time to disease onset, our simulation results show that, in the presence of a cure fraction, both types of analysis can be sensitive to either type of effect. When performing a genome-wide Cox PH analysis to identify genetic variants that affect time to disease onset, the estimated hazard ratios may be inflated if the genetic variants also affect disease risk. In fact, if genetic variants only influences disease risk, but not age of onset, a traditional survival analysis may erroneously conclude that an age of onset effect is present. Moreover, performing a logistic analysis to estimate the genetic effect on disease risk is even more problematic, since logistic analysis does not take into account the censoring typically observed in survival data. Our results indicate that in this context the power of logistic analysis to detect a logistic effect is even lower than that of Cox PH analysis. In summary, when a cure fraction is present both logistic regression and Cox PH regression result in biased estimates of genetic effect sizes. Note that due to model misspecification it is to be expected that logistic analysis or Cox survival analysis are suboptimal when applied to cure model data. However, our results show that under a cure model the traditional Cox survival model is not just biased, but that it can identify age of onset effects that do not exist. Whether or not a cure model is applicable depends on the genetic architecture of the data at hand. Some genetic variants may influence either disease risk or age of onset, while others may influence both.

Our second aim was to investigate whether the cure model offers advantages over traditional survival analysis. We have applied a relatively unknown model that allows for the estimation of both logistic and survival effects: the cure survival model. Under a cure model, cure survival analysis leads to unbiased estimates, but only in the absence of background genetic risk. It is not surprising that survival data simulated with a cure survival model is best analyzed with a cure survival analysis, as our results imply. So why not always use a cure survival analysis if we suspect a cure fraction? Unfortunately, the cure survival mixture model is computationally too demanding to apply at a genome-wide scale. A running time of 25 s per SNP for analyzing 5 million SNPs would amount to 1446 computing days on a single core for only 500 subjects. This is prohibitively long for most GWA studies. Furthermore, the logistic effect of the cure model is unidentified if the cure fraction is zero or one and therefore if the cure fraction is close to either boundary the running time increases further or even worse, the algorithm does not converge. On the other hand, the above running times do allow a post hoc analysis of a limited number of potentially interesting findings. Similarly, cure survival analysis can be used in candidate gene or replication analyses, in which a relatively small number of genetic variants are typically tested.

Although cure survival analysis removes bias in parameter estimation due to cure fractions, it does not remove bias due to genetic background risk. This latter bias also occurs in traditional case–control GWA studies (Gail et al. 1984; Stringer et al. 2011). Since large amounts of background heritability is typical in complex diseases (Gratten et al. 2014), cure analysis will result in biased estimates of both the logistic and the survival effect. Our results suggest that a cure survival analysis will underestimate both effects and that this underestimation is relatively independent from the cure fraction. Although the odds ratio of a cure survival analysis is less biased than that of a traditional logistic regression, its hazard ratio is more biased than that of a Cox PH regression.

In many genome-wide survival studies of mental or physical disorders, we do not expect that all subjects will eventually develop the disease of interest. Based on our results, we therefore provide three recommendations for genetic analysis of diseases or other phenotypes that will not affect the entire population.

Our first recommendation is to follow-up significant hits resulting from a Cox PH analysis with a cure PH analysis. Cure survival analysis allows separation of genetic variants which primarily influence the time of disease onset from those variants that primarily influence disease risk. Distinguishing these two types of genetic variants may provide valuable insight into the biological processes behind a disease. However, as our results suggest, cure survival analysis should be only considered if the cure fraction in the sample is sufficiently large (e.g. >0.1), but not too large (e.g. <0.9). This means for a population cohort that the disease of interest should have a lifetime prevalence between 10 and 90 %. Since most disease prevalences are <10 %, sampling from a high-risk population could be considered to decrease the cure fraction in the sample. Although in principle cure analysis is likely to improve prediction of both disease risk and age of onset in independent samples under a cure model, in practice outcome prediction using a small number of genetic variants is difficult in complex traits that are influenced by many variants across the genome.

We also recommend that if distinguishing the type of genetic effect (logistic vs survival) is not a primary concern, a genome-wide Cox PH analysis should be used to maximize the power to detect either type of genetic effect. Our results show that the power of a Cox PH analysis to detect either type of genetic effect is larger than that of the cure survival analysis.

Our third recommendation is that power analyses of genome-wide survival studies should account for cure fraction, logistic and survival effect sizes, and background heritability. Ignoring these factors will greatly influence the estimated power. A reasonable assumption for the cure fraction is 1 – lifetime prevalence. Effect sizes are difficult to predict in advance and ideally power is calculated for a range of potential effect sizes for both the logistic and the survival effect. Finally, for complex diseases it is safe to assume that the background heritability will be close to the disease heritability. Although general power calculators for survival analysis exist, such as the genetic power calculator for case–control and quantitative trait GWA studies (Purcell et al. 2003), we are not aware of programs or web tools that are tailored towards genome-wide survival studies. However, power analysis through a simplified simulation of GWA data is a viable alternative as exemplified by this simulation study.

The results of this simulation study should be interpreted in the context of two limitations. First, it was not computationally feasible to vary all relevant model parameters. For example, we used a small sample of 500 subjects in all our simulations and investigated only a limited number of combinations of effect sizes. Second, data were simulated according to a single distribution: the Weibull distribution. In reality, survival data may be distributed differently. The implications and recommendations of this study are therefore more qualitative than quantitative in nature.

The primary focus of this study was to investigate the impact of cure fractions and genetic background risk on bias in genome-wide survival analysis. However, other factors may introduce bias in a genome-wide survival analysis. For example, the assumption of uninformed censoring may not hold, resulting in underestimated or overestimated hazard ratios depending on the type of informed censoring. Similarly, a GWA sample may include multiple family members of a family. In that case sandwich estimation can be used to account for the correlations caused by family relations within a sample (Borecki and Province 2008; Diggle et al. 2002). This type of estimation increases the standard error of the estimate to correct for dependence due to familial relatedness. Studying the effects of different types of censoring and presence of familial relatedness on bias and power was beyond the scope of this study, but these effects warrant further research.

In conclusion, cure fractions introduce the possibility of investigating the impact of both logistic and survival effects of a single genetic variant. Cure survival analysis takes this complexity into account. Although the application of cure survival analysis is not feasible at a genome-wide scale, we have shown that follow-up of a specific subset of SNPs may provide information about logistic and survival effects in a relatively unbiased way. Distinguishing genetic variants affecting disease onset and those affecting disease risk is an important step in understanding the nature of genetic effects in (neuro)psychiatric disorders.
